# Bridging the clinical gap: Confidence informed IDH prediction in brain gliomas using MRI and deep learning

**DOI:** 10.1093/noajnl/vdaf142

**Published:** 2025-07-25

**Authors:** Chandan Ganesh Bangalore Yogananda, Nghi C D Truong, Benjamin C Wagner, Yin Xi, Jason Bowerman, Divya D Reddy, James M Holcomb, Niloufar Saadat, Kimmo J Hatanpaa, Toral R Patel, Baowei Fei, Matthew D Lee, Rajan Jain, Richard J Bruce, Ananth J Madhuranthakam, Marco C Pinho, Joseph A Maldjian

**Affiliations:** Department of Radiology, UT Southwestern Medical Center, Dallas, Texas, USA; Department of Radiology, UT Southwestern Medical Center, Dallas, Texas, USA; Department of Radiology, UT Southwestern Medical Center, Dallas, Texas, USA; Department of Radiology, UT Southwestern Medical Center, Dallas, Texas, USA; Department of Radiology, UT Southwestern Medical Center, Dallas, Texas, USA; Department of Radiology, UT Southwestern Medical Center, Dallas, Texas, USA; Department of Radiology, UT Southwestern Medical Center, Dallas, Texas, USA; Department of Radiology, UT Southwestern Medical Center, Dallas, Texas, USA; Department of Pathology, UT Southwestern Medical Center, Dallas, Texas, USA; Department of Neurological Surgery, UT Southwestern Medical Center, Dallas, Texas, USA; Department of Bioengineering, UT Dallas, Richardson, Texas, USA; Department of Radiology, NYU Grossman School of Medicine, New York, New York, USA; Department of Neurosurgery, NYU Grossman School of Medicine, New York, New York, USA; Department of Radiology, NYU Grossman School of Medicine, New York, New York, USA; Department of Radiology, University of Wisconsin School of Medicine, Madison, Wisconsin, USA; Department of Radiology Mayo Clinic, Rochester, Minnesota, USA; Department of Radiology, UT Southwestern Medical Center, Dallas, Texas, USA; Department of Radiology, UT Southwestern Medical Center, Dallas, Texas, USA

**Keywords:** brain glioma, CNN, confidence score, deep learning, IDH classification

## Abstract

**Background:**

The isocitrate dehydrogenase (IDH) mutation status is a key molecular marker in diagnosing and treating brain tumors. Currently, it is determined via invasive tissue biopsy. Recent advances in deep learning (DL) have offered promising non-invasive alternatives for determining IDH status. However, their clinical translation is hindered by a significant gap between DL predictions and their clinical applicability. The limited transparency of many DL-networks and inadequate evaluation metrics hinders trust and adoption, as clinicians require clear and validated insights for determining IDH status. These challenges highlight the need for robust validation and measures of predictive reliability to make DL-predictions clinically actionable.

**Methods:**

We developed a unique approach for non-invasive prediction of IDH status using MRI. We combine a voxel-wise-segmentation network(MC-net) with Bayesian logistic regression (BLR) to provide an IDH status and estimate confidence scores. We utilized a comprehensive dataset of 2,481 glioma cases from eight institutions.

**Results:**

Our framework(MC-net + BLR) demonstrated robust performance achieving 96.4% and 95.1% classification accuracies on diverse databases, with an AUC of 0.98. The BLR was implemented exclusively on held-out test data, ensuring that the derived confidence scores are independent of the training or validation phases. The derived confidence scores showed a low Brier score of 0.0125, highlighting its superior calibration and uncertainty quantification.

**Conclusion:**

The developed framework provides an IDH status and a confidence score, offering clinicians an additional layer of assurance in prediction reliability. It bridges the gap between high-performing DL models and their clinical applicability by addressing the challenges in prediction reliability. Our framework is a significant advancement in non-invasive determination of IDH-status and confidence-informed therapeutic decision-making in neuro-oncology.

**Key Points**

IDH status is an important genetic marker for gliomas

We developed a noninvasive, MRI-based deep-learning method for predicting IDH status and providing a confidence score for each prediction.

**Importance of the Study**

Isocitrate dehydrogenase (IDH) mutation status is a critical molecular marker for diagnosing and treating brain tumors. Traditionally, the IDH status is determined via an invasive brain biopsy, which may not always be feasible—particularly in surgically inaccessible tumors. The ability to non-invasively obtain IDH status has significant implications in determining therapy and prognosis. It enables better surgical decision-making, risk assessment, and the potential to guide neoadjuvant or targeted therapies before biopsy or resection. Recent advances in deep learning (DL) have shown great promise in determining IDH status noninvasively. However, their clinical translation is hindered due to a significant gap between DL predictions and their clinical applicability. To address these limitations, this study introduces a unique DL framework that combines a voxel-wise segmentation network (MC-net) with Bayesian logistic regression (BLR) for noninvasive prediction of IDH status using MRI. The framework achieves high classification accuracy across diverse imaging protocols and provides reliable confidence scores, bridging the gap between computational advances and clinical utility. This work represents a transformative step toward non-invasive, reliable, and actionable glioma characterization, with the potential for imminent clinical translation in neuro-oncology.

Gliomas are the most common type of primary malignant brain tumor. They present significant challenges in clinical diagnosis and treatment. An important discovery in brain tumor biology is the identification of isocitrate dehydrogenase (IDH) mutation status as a marker for therapy and prognosis.^1^ Traditionally, IDH status is determined through immunohistochemistry or gene sequencing on a tissue specimen acquired through brain tissue sampling. Surgical resection remains a central component of standard-of-care management for most diffuse gliomas, enabling both histopathological diagnosis and maximal safe tumor removal. However, biopsy samples may not always yield enough tumor tissue for comprehensive molecular characterization.^2^ Additionally, brain tissue sampling can be challenging in deep-seated tumors with poor functional status, making noninvasive molecular prediction a valuable diagnostic alternative. While histopathology remains the gold standard, noninvasive methods for early molecular characterization—such as IDH prediction—are valuable adjuncts for preoperative planning. With the advent of the new IDH inhibitor drugs, neoadjuvant medical approaches may become a reality and could be informed by non-invasive determination of IDH status. Therefore, there is a need to develop a robust and reliable noninvasive approach for determining the IDH status.

Recent advances in deep learning (DL) and medical imaging have offered promising noninvasive alternatives for glioma characterization.^3,4^ Several deep learning networks (DLNs) have demonstrated substantial success in various medical imaging tasks, including molecular profiling of brain tumors.^3,5–14^ These networks can analyze complex patterns in MR images to predict molecular and genetic characteristics, such as IDH mutation status. Despite their high accuracy, clinical translation of these models is often hindered by a significant gap between DL predictions and their clinical applicability.^15,16^ This gap is primarily due to traditional evaluation metrics (accuracy, precision, and recall) failing to adequately capture the reliability and confidence of the DL predictions in a clinical setting.^16,17^

DLNs are prone to overfitting, especially when trained on limited or non-representative datasets. This can lead to DLNs performing exceptionally well during the developmental phase but poorly on unseen clinical data. This limits their generalizability and robustness.^18^ Additionally, the variability in imaging protocols and equipment across institutions can affect the consistency and reliability of DLN predictions.^19^ Another challenge in translating DL predictions to clinical practice is the limited transparency in DL models.^15–17^ Clinicians often require a reliable DL prediction, especially in critical decisions such as determining IDH mutation status. However, the limited transparency of many DLNs has highlighted the need to quantify prediction reliability to support clinical trust and adoption.^15,16^ Furthermore, traditional evaluation metrics fall short in providing comprehensive insights into DLN’s performance across diverse databases and imaging conditions, crucial for clinical adoption.^16,17^ These factors contribute to the hesitation among clinicians to rely solely on DL predictions without additional validation and robust testing frameworks.^18,19^

To address these limitations, we propose a unique deep learning framework to predict IDH mutation status using MR images and provide a confidence score (CS) for each prediction. The developed framework includes a U-Net architecture for voxel-wise segmentation and classification, combined with Bayesian logistic regression (BLR) to estimate confidence scores. This confidence-informed approach aims to enhance the clinical relevance of DL predictions. It offers a tangible measure of DL predictions, aiding clinicians in making more informed decisions. The proposed method can bridge the gap between high-performing DLNs and their clinical utility.

## Material and Methods

### Database

Brain tumor MRI and genomic information were obtained from five publicly available databases and three in-house/collaborator institutions. The public databases included The Cancer Imaging Archive (TCIA),^20–22^ Erasmus Glioma Database (EGD),^23^ University of California San Francisco Preoperative Diffuse Glioma MRI dataset (UCSF),^24^ University of Pennsylvania Health System (UPenn),^25^ and IvyGAP dataset.^22^ The collaborator institutions included UT Southwestern Medical Center (UTSW-1, UTSW-2, and UTSW-3), New York University (NYU), and University of Wisconsin-Madison (UWM). Subjects were screened for the availability of IDH mutation status and multi-contrast MR images. Only pre-operative studies were included in the dataset. A breakdown of tumor grade, presence of enhancement, and IDH Status of all datasets included in the study is as shown in Table 1. This study was approved by the Institutional Review Board at UTSW.

**Table 1.** A Breakdown of Tumor Grade, Presence of Enhancement, and IDH Status of All Datasets Included in the Study

	TCIA	Ivy-GAP	UCSF	EGD	Upenn	UTSW-1	UTSW-2	UTSW-3	NYU		UWM	TOTAL
**Mutant**	92	2	103	150	11	106	22	28	47		16	**577**
**Wildtype**	112	21	392	306	387	254	37	77	130		188	**1904**
**TOTAL**	204	23	495	456	398	360	59	105	177		204	**2481**
											
	Training Data (TCIA, UTSW1, UCSF)					Testing data (NYU, UWM, EGD, UPenn, UTSW2, UTSW3)						TOTAL
Tumor Grade	IDH Mutated		IDH wildtype			IDH Mutated		IDH wild type				
**Grade 2**	142		24			157		22				**345**
**Grade 3**	106		55			54		19				**234**
**Grade 4**	48		672			21		470				**1211**
**Grade Unavailable**	3		31			42		642				**690**
**TOTAL**	299		782			274		1126				**2481**
	**Training Data** ** (TCIA, UTSW1, UCSF)**					**Testing data** **(NYU, UWM, EGD, UPenn, UTSW2, UTSW3)**						**TOTAL**
**Presence of Enhancement**	**Enhancing**		**Non-Enhancing**			**Enhancing**		**Non-Enhancing**				
	**IDH Mutated**	**IDH wildtype**	**IDH Mutated**		**IDH wildtype**	**IDH Mutated**	**IDH wildtype**	**IDH Mutated**		**IDH wildtype**		
**Grade 2**	37	8	105		16	45	8	112		14		**345**
**Grade 3**	66	31	40		24	21	7	33		12		**234**
**Grade 4**	48	657	0		15	20	445	1		25		**1211**
**Grade Unavailable**	3	28	0		3	29	602	13		13		**691**
**TOTAL**	154	724	145		58	115	1062	159		64		**2481**

### Training and Testing Data

- A combination of TCIA, UTSW-1, IvyGAP, and UCSF datasets was used for training (1088 cases).
- The testing cohort was split into two parts, TestGroup-1 (TG-1) and TestGroup-2 (TG-2).
- TG-1 consisted of MRIs from NYU, UWM, EGD, and UPenn (1236 cases).
- TG-2 consisted of MRIs from UTSW-2 and UTSW-3 (164 cases).
- Both TG1 and TG2 were true held-out test cases.

### Pre-processing Steps

Multi-contrast native space MRI (T1-pre, T1-post, T2-w, and T2-FLAIR), from TCIA, IvyGAP, UTSW-1, UTSW-2, UTSW-3, NYU, UWM, and UPenn were pre-processed using the FeTS platform.^26^ FeTS co-registers the images to the SRI24 template space,^27^ performs skull-stripping, and multi-class brain tumor segmentation. MRI from the UCSF database was readily available as pre-processed images (co-registered and skull-stripped).^24^ The EGD data were also readily available as pre-processed images (co-registered to MNI152 space). The EGD data were further skull stripped using the Advanced Normalization Tools (ANTS).^23,28^ Additionally, all datasets were N4 bias corrected and z-score normalized.^29,30^

**Figure 1.**
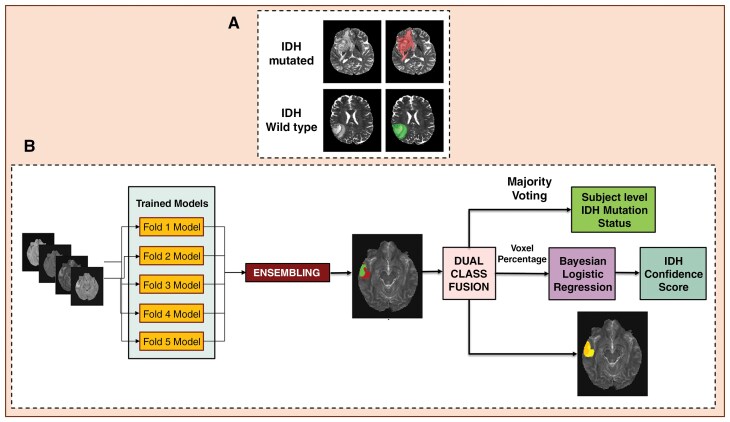
(**A)** Ground truth whole tumor masks. Red voxels represent IDH mutated (values of 1), and green voxels represent IDH wild type (values of 2). The ground truth labels have the same mutation status for all voxels in each tumor. (**B)** Represents the Testing procedure to obtain IDH mutation status and Confidence Score. The Fold boxes represent the networks from individual folds, where the MRI is tested. Majority voting across voxels is used to determine the overall IDH mutation status.

Multi-class FeTS tumor segmentations were combined to generate whole-tumor masks. The generated Whole-tumor masks for IDH-mutated and IDH wild-type cases were labelled with 1 and 2, respectively (Figure 1). These tumor masks were used as the ground truth in the training step. IDH wildtype was used as the positive class in our study.

### Network Implementation and Cross-validation

A multi-contrast 2D network (***MC-net***) was developed using pytorch.^31^*nnUNet*^32^ was used for its robust preprocessing, data augmentation strategies, and standardized training pipelines. MC-net model architecture was developed in-house.^3^ Network architecture and hyperparameters were chosen based on our prior work with IDH (Supplementary Table 2).^3^ A 5-fold training-only Cross-Validation (CV) procedure was implemented to train ***MC-net***. The training data (TCIA + UTSW-1 + IvyGAP + UCSF) was randomly shuffled and split with 80% used for training and 20% used for in-training validation (model optimization). The networks were trained for a voxel-wise dual-class segmentation of the whole tumor with Classes 1 and 2 representing mutated and wild-type, respectively.^33^ To avoid the problem of *data leakage*, data shuffling and splitting were implemented at the subject level.^34,35^ To generalize the model’s performance, the training data were reshuffled during each fold of the cross-validation procedure. Each fold in the CV procedure represents a new training phase on a unique combination of the dataset. The optimized Hyperparameters are provided in *Supplementary Table S1*. Once the CV procedure was completed, the trained models were evaluated on the true held-out test cases (***TG-1*** and ***TG-2***).

### Testing Procedure

Output segmentations from each model of the CV procedure were ensembled to obtain the final two-class segmentation volume. A majority voting over the voxel-wise classes of IDH-mutated or IDH-wild-type provided an IDH classification for each subject. Networks were implemented on Tesla A100 NVIDIA-GPUs. The IDH classification process is fully automated, and the tumor segmentation is a natural output of the voxel-wise classification approach.

### Estimating Confidence Scores

- ***Testing TG-1:*** The trained MC-net was tested on TG-1. The network segments the whole tumor voxels as either IDH mutated or IDH wild type. For each subject in TG-1, the percentage of IDH-wildtype voxels was obtained by dividing the predicted number of IDH-mutated voxels by the total number of predicted voxels in each tumor (mutated voxels + wild type voxels).
- ***Curve fitting with TG-1:*** The predicted mutated voxel percentages and the corresponding ground truth data from TG-1 were used to fit a Logistic Regression (LR) model (Eqn.1) and a Bayesian logistic regression (BLR) model (Eqn.2).

A Logistic Regression (LR) was chosen for its simplicity in modeling the relationship between predicted wildtype voxel percentages and ground truth IDH mutation status. A Bayesian Logistic Regression (BLR) was chosen for its ability to quantify uncertainty in the predictions and to capture both linear and non-linear relationships between voxel percentages and the ground truth labels, offering a more robust method for estimating confidence scores.

The logistic function used in modeling both LR and BLR was kept constant (Eqn. 1).


$$\begin{array}{l} P(y=1\;\;|\;x,\;\alpha ,\beta )=\frac{1}{1+\exp\left(-\left(\alpha +\beta .\;x\right)\right)} \end{array}$$ (1)

#### Logistic regression (LR)

- *α* is the intercept (bias term).
- *β* is the coefficient (weight) for the input feature *x.*
- *x* represents the *input feature* (*wildtype_voxel_percentage*).
-
exp(−(α+β. x)) converts the linear combination of inputs into a non-linear function.

#### Inputs

- o*x→wildtype_voxel_percentage*.
- o*y→* Ground truth label (binary: 0 or 1)
- o*α*, *β:* Parameters estimated during curve fitting

*Outputs:*

- oP(y=1  | x, α,β): The Confidence Score on ***MC-net***’s prediction.

#### Bayesian logistic regression (BLR)

In Bayesian logistic regression, *α* and *β* are treated as random variables with prior distributions.

- *α* and *β* are sampled from prior distributions.
- *α ~* Normal(0, σ^2^)
- *β ~* Normal(0, σ^2^)

The posterior distribution of *α* and *β* is computed after observing the data using:


$$\begin{array}{l} P(\alpha ,\beta \;\;|\;\;x,y)\propto P(y\;\;|\;x,\;\alpha ,\beta ).\;P\left(\alpha \right).\;P\left(\beta \right)) \end{array}$$ (2)

*Inputs:*

- o*x→wildtype_voxel_percentages*.
- o*y→* Ground truth label (binary: 0 or 1)
- o*α*, *β:* Parameters with priors (e.g. *α*, *β ~* Normal(0, σ^2^))

*Outputs:*

- o Posterior distributions for *α* and *β.*
- o Confidence Scores P(y=1  | x, α,β): computed using posterior samples of *α* and *β*.
- *An informative prior* was defined as a Gaussian prior with mean = 0 and variance = 1, ie, N(0,1). It incorporates a moderate degree of prior belief that the weights of the logistic regression model are likely to lie close to zero. It encourages the model to favor simpler explanations and provides regularization during confidence calibration.
- *A non-informative prior* was defined as a Gaussian prior with mean = 0 and variance = 1000, ie, N(0,1000). It represents minimal prior knowledge or constraints. This allows the posterior to be influenced almost entirely by the observed data, effectively approaching a maximum likelihood estimation scenario.
- These priors were chosen to explore how different parameter uncertainty affects the calibration of voxel-percentages into subject-level confidence scores.
- ***Testing TG-2:*** The TG-2 dataset was then tested using the trained ***MC-net*** to predict the wildtype voxel percentages for each subject.
- ***Confidence Score Estimation:*** The ***LR*** and ***BLR models*** (*after curve fitting—*Figure 3) were used as a reference (separately) to estimate confidence scores for the predicted voxel percentages in ***TG-2*** subjects. This step provided a confidence score for each prediction.
- ***Evaluating the Confidence Score:*** To ensure an unbiased assessment, the estimated confidence scores on TG-2 (held-out test data) were evaluated using the Brier score method.

**Figure 2.**
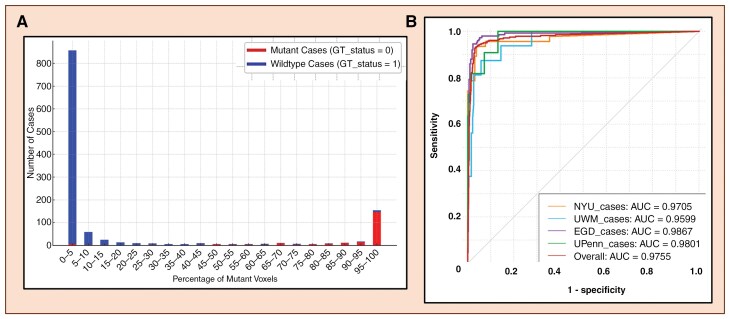
**(A)** Bi-modal Distribution of percent mutant voxels on TG-1. The distribution shows a clear differentiation between mutated and wildtype gliomas with high voxel percentage predictions of mutation status for each class. **(B)** ROC and AUC Performance (subject-wise) of MC-net on individual databases in TG-1.

**Figure 3.**
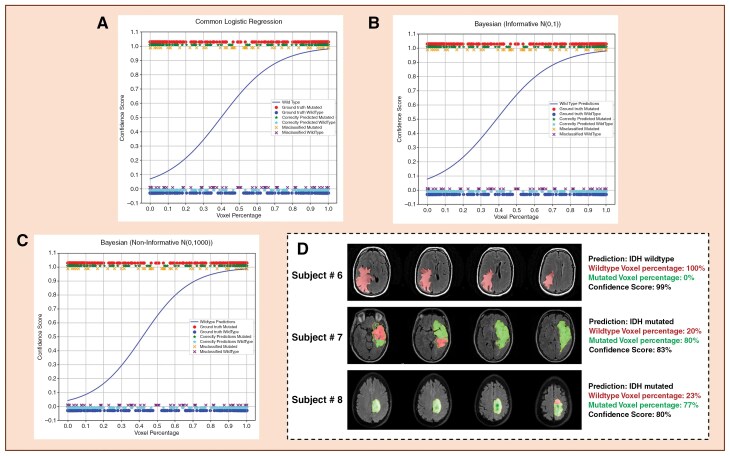
Represents the different curve fitting methods used to estimate confidence scores with a few example scenarios. **(A)** Logistic Regression, **(B)** Bayesian Logistic regression with informative priors, **(C)** Bayesian Logistic regression with non-informative priors, **(D)** Examples of estimated confidence scores. The location of the dots along the x-axis (3A–3C) represents a single test subject from the TG-1, and the dot color and marker type indicate (i) Ground-truth label (red or blue dot for mutant or wildtype), (ii) Correct vs. incorrect classification (* for correct, x for incorrect), and (iii) Class-specific error type (eg, misclassified mutant vs. wildtype).

This approach eliminates the potential influence of training or validation data, providing a true measure of the model’s reliability and generalizability.

## Results

### IDH Classification on Test Group 1 (TG-1)

The MC-net demonstrated high classification performance on TG-1, which included datasets from NYU, UWM, EGD, and UPenn (Table 2, Supplementary Fig. 1, Supplementary Table 1). The network achieved an overall accuracy of 96.4%, with dataset-specific accuracies ranging from 93.6% (UWM) to 98.0% (UPenn). The network also achieved an overall AUC of 0.98, underscoring its robustness in distinguishing IDH-mutated and IDH wildtype gliomas. The precision was 98.2%, and the recall was 97.3%, ensuring consistent performance in classifying the mutation status.

**Table 2.** Represents the Performance of MC-net on Both Test Groups (TG-1 & TG-2).

IDH Classification accuracy on *Test group-1*						
Training Dataset	Metrics	NYU (177)	UWM (204)	EGD (456)	UPenn (399)	Overall Accuracy
		(47/130)	(16/188)	(150/306)	(11/388)	
**TCIA +** **UTSW-1 + IvyGAP + UCSF** 1088 Subjects (301 / 787)	Accuracy	**96.0**	**93.6**	**96.3**	**98.0**	**96.4**
	Accuracy (IDH-mut class)	93.6	81.2	93.3	81.8	92.0
	Accuracy (wildtype class)	96.9	94.7	97.7	98.5	97.3
	Precision	97.7	98.3	96.8	99.5	98.2
	Recall	96.9	94.7	97.7	98.5	97.3
	F1-score	97.3	96.5	97.2	99.0	97.8
	AUC	0.97	0.96	0.98	0.98	0.98

	**IDH Classification accuracy on *Test group-2***					
	-	-	-	**UTSW-2 (59)**	**UTSW-3 (105)**	**Overall Accuracy**
	-	-	-	(22 / 37)	(28 / 77)	
	Accuracy	-	-	93.2	96.2	**95.1**
	Accuracy (IDH-mut class)	-	-	95.5	96.4	96.0
	Accuracy (wildtype class)	-	-	91.9	96.1	94.7
	Precision	-	-	97.1	98.6	98.2
	Recall	-	-	91.9	96.1	94.7
	F1-score	-	-	94.4	97.3	96.4
	AUC	-	-	0.98	0.97	0.98

Figure 2 shows the bimodal distribution of voxel percentages in TG-1. This distribution demonstrates a clear separation between IDH-mutated and wildtype gliomas, further validating the reliability of voxel-wise predictions. The high voxel percentage predictions for each class align well with their respective mutation status, emphasizing MC-net’s accuracy. Figure 2 also presents the ROC curves (subject-wise) for the TG-1 datasets, highlighting the network’s capability in achieving excellent classification across multiple datasets from different institutions.

### IDH Classification on Test Group 2 (TG-2)

Testing group TG-2 (UTSW-2 and UTSW-3 cohorts) further demonstrated the generalizability of MC-net. As shown in Table 2, the network achieved an overall accuracy of 95.1%. Precision was maintained at 98.2%, and recall at 94.7%, underscoring the network’s consistent ability to accurately classify IDH mutation status. These results highlight the robustness of MC-net in handling unseen data, effectively accounting for variability in imaging protocols and institutional differences and reinforcing its potential for broader clinical application.

### Confidence Score Estimation

Figure 3 illustrates the different curve-fitting methods used for obtaining confidence scores (LR and BLR methods). The estimated confidence scores (Table 3) frequently exceeded the overall specificity. For instance, in IDH-wildtype cases, the BLR model (with non-informative priors) estimated consistently higher confidence levels than the achieved specificity. This observation highlights the model’s ability to reliably quantify its predictive certainty.

**Table 3.** **Brier Scores** for the Different Regression Curves and Examples of Estimated Confidence Scores.

				Using raw wildtype voxel percentages	Logistic Regression	Bayesian Logistic Regression Informative Priors N(0,1)	Bayesian Logistic Regression Non- Informative Priors N(0,1000)
			**Brier Score** **(squared error)**	**0.0435**	**0.0158**	**0.0150**	**0.0125**

**Examples** **(Subject No.)**	**Ground truth IDH Status**	**Predicted IDH status**	**Predicted mutated voxel percentage**	**Predicted wildtype voxel percentage**	**Example—Estimated Confidence Scores**		
					**Logistic Regression**	**Bayesian Logistic Regression (N(0, 1))**	**Bayesian Logistic Regression (N(0, 1000))**
**Subject #1**	Mutated	Mutated	99.4	0.06	0.914	0.919	0.931
**Subject #2**	Mutated	Mutated	85.6	15.4	0.861	0.872	0.894
**Subject #3**	Wildtype	Wildtype	0.9	99.1	0.979	0.989	0.994
**Subject #4**	Wildtype	Wildtype	19	81	0.932	0.958	0.972

Table 3 provides the Brier Scores for each regression method. BLR with non-informative priors achieved the lowest Brier Score, indicating superior calibration and uncertainty quantification. The curves in Figure 3 demonstrate the smooth fit between predicted voxel percentages and ground truth labels, showcasing the ability of Bayesian methods to provide robust confidence estimates.

## Discussion

We developed and evaluated an MRI-based deep-learning framework (MC-net + BLR) to predict IDH mutation status and provide confidence scores. This study demonstrates the potential of combining DL predictions with confidence scores to enhance clinical applicability. It bridges the gap between DL predictions and clinical relevance, emphasizing the value of reliability measures in IDH classification. The developed framework achieved high classification accuracy on datasets from multiple institutions, showcasing its ability to handle diverse imaging conditions and institutional variability. Furthermore, the comparative evaluation of LR and BLR models to estimate confidence scores highlights the importance of robust uncertainty quantification. These factors underscore the advantages of the developed framework over existing IDH classification algorithms.

***MC-net*** achieved high accuracy, sensitivity, and specificity across TG-1 and TG-2, demonstrating its robustness and ability to generalize across diverse institutional datasets. The ROC curves (Figure 2) and the bimodal voxel percentage distributions (Figure 2) further underscore the network’s capacity to distinguish between IDH-mutated and wild-type gliomas reliably. These findings establish MC-net as a dependable network for clinical application, particularly in scenarios requiring noninvasive tumor characterization. The addition of confidence scores significantly enhances the reliability of MC-net’s predictions. It provides clinicians with a quantifiable measure of prediction reliability, critical for cases involving high uncertainty. The voxel-wise classification of MC-net can lead to parts of the tumor labelled as either IDH mutated or IDH wildtype. However, we do not claim that MC-net is able to detect heterogeneity in IDH mutation status. Rather, the mixed classification results likely reflect the morphologic heterogeneity (imaging expressions) of the IDH mutation status within a given tumor.

Confidence scores were estimated on TG-2 using raw wildtype voxel percentages, LR, and BLR models. The obtained scores were evaluated using the Brier score method,^36^ ensuring an unbiased assessment of the model’s calibration. The Brier Score analysis (Table 3) shows that the BLR with non-informative priors (N(0,1000)) demonstrated superior performance in estimating confidence scores. This superior performance can be attributed to its ability in capturing uncertainty more flexibly, especially in scenarios where the data provides the majority of the information for parameter estimation.^37^ Non-informative priors impose minimal assumptions on the parameter distributions, allowing the model to rely predominantly on the observed data rather than being constrained by pre-defined beliefs or biases.^38^ This is particularly advantageous as the voxel percentage distributions can exhibit distinct yet complex patterns, as evidenced by the bimodal distribution in TG-1. By leveraging a non-informative prior, the BLR model can adapt more effectively to variations within the data, improving its calibration and reducing overconfidence in predictions.^39^

The addition of confidence scores significantly enhances the predictive reliability of MC-net. Using non-informative BLR is highly beneficial as it aligns with minimizing unwarranted bias in decision-making. The reduced Brier Score (0.0125) indicates superior calibration and excellent correlation between MC-net predictions and estimated confidence scores. This robustness enables clinicians to rely on the network’s predictions, particularly in cases where tumor location poses a high risk of postoperative complications. Additionally, the flexibility of non-informative priors makes this approach generalizable to diverse datasets, enhancing its utility across institutions with varying imaging protocols. This makes non-informative BLR a strong choice for clinical integration, where DL predictions need to be reliable.

The smooth curve fitting observed in Figure 3 demonstrates BLR’s ability to handle non-linear relationships between voxel percentages and ground truth labels. An interesting trend in the wildtype curves (Figure 3) is that the estimated confidence scores (Table 3) can exceed the overall prediction accuracy for the wildtype class (specificity). This can be attributed to the distinct voxel percentage patterns, enabling BLR to estimate with greater certainty. It also demonstrates that confidence scores can serve as a reliable measure of predictive certainty, offering an additional layer of assurance for clinicians relying on DL predictions. This ability to effectively capture variability in voxel percentages and provide confidence scores further validates its clinical utility. These insights provide opportunities to prioritize cases with higher predictive confidence, streamlining the decision-making processes in clinical workflows.

The developed framework demonstrates superior performance compared to several existing machine learning and deep learning algorithms. Zhang et al. utilized multimodal MRI features combined with radiomics and random forest to detect IDH mutations, achieving an accuracy of 86% and 89% in training and validation, respectively.^6^ Hosseini’s (2023) study employing radiomic features from core tumor subregions achieved an AUC of 0.93, but was limited by small sample size (*n* = 57) and reliance on semi-automated segmentation workflows.^13^ Zhang et al. (2023) combined conventional radiomics and deep learning to predict IDH status, reporting an AUC of 0.92 on single-institution data, limiting generalizability.^14^ Chang et al. utilized CNNs on MR images, achieving an accuracy of 94% but lacked robustness across datasets with diverse imaging protocols and did not describe accounting for data leakage in the model.^7^ Cluceru et al. (2022) developed a CNN using diffusion-weighted and anatomical MRI, achieving a 95% test accuracy for IDH mutation status and 85.7% for a three-class classification involving IDH and 1p19q. While the test cohort (*n* = 147) was of moderate size, it was limited to a single institution, and the model’s reliance on diffusion-weighted imaging may limit its generalizability across diverse clinical settings.^9^ Pasquini et al. developed a GBM-specific CNN model, achieving a maximum accuracy of 83% using rCBV maps, but the limited patient cohorts restrict generalizability.^40^ Similarly, the hybrid approach by Choi et al., while achieving accuracies of up to 93.8% on internal test sets, displayed a significant drop in external validation accuracy (78.8%), revealing potential overfitting and susceptibility to inter-institutional variability.^41^ Similarly, Wu et al.’s (2024) Swin transformer averaged an AUC of 0.96 on the internal test data, while the AUC dropped significantly (0.842) on the external test.^8^ Liu et al. developed a deep learning model to predict IDH status from histopathology images, achieving an accuracy of 88%.^42^ However, the focus on histopathology images means that the effectiveness of the model is contingent on the availability and quality of such images, which vary significantly across different healthcare facilities. Decuyper et al. utilized a U-Net for segmentation followed by a CNN for classification, reporting accuracies of 94% and 86% on internal and external datasets, respectively.^43^ However, their dependence on brain tumor segmentation can introduce potential variability and inefficiencies in clinical workflows. Manikis et al. highlighted the importance of explainability in radiomics models but noted a lack of consistent external validation.^44^ Additionally, several previous studies, including Zhang et al., relied heavily on traditional radiomic features or single-center datasets.^6^ These limitations hinder generalizability and clinical adoption. Furthermore, the reliance on specific imaging protocols restricts model deployment across diverse clinical settings. Leveraging diverse datasets from multiple institutions, our approach mitigates these concerns, ensuring broader applicability. Our algorithms employ subject-level data splitting to mitigate the problem of data-leakage commonly found in slice-wise methodologies.^34,35^ Our work overcomes the limitations of previous studies by evaluating MC-net on large, diverse, true held-out datasets, yielding more reliable accuracy estimates. Furthermore, the estimated confidence score represents a tangible measure of the MC-net’s reliability in predicting IDH-status. These comparisons highlight the advancements of our framework in bridging the gap between research and clinical application.

Conventional deep learning models produce softmax probabilities reflecting the model’s prediction as learned from the training distribution. However, these probabilities are point estimates derived from a deep learning model and may not accurately capture uncertainty when applied to out-of-distribution or held-out test data. This often leads to overconfident or mis-calibrated predictions. To address this issue, we implemented BLR exclusively on a held-out test data (TG-1), ensuring that the confidence scores derived are independent of the training and validation phases. The key contribution of the developed framework is that it provides a quantitative measure of confidence on the subject-level prediction by modeling the relationship between voxel percentages and true IDH status using BLR. This is a notable advantage over previous studies, which often optimize and assess their networks using validation data. Such practices risk introducing biases or overestimating the model’s generalizability. This method enables clinicians to assess the reliability of DL outputs, crucial in clinical contexts where overconfident misclassifications may lead to inappropriate treatment decisions. The confidence score provides a reliable measure of model trustworthiness, fostering clinician confidence and facilitating the integration of deep learning predictions into routine clinical workflows. Furthermore, the inclusion of a second independent held-out test dataset (TG-2) reinforces the robustness of our method, confirming its ability to generalize across diverse imaging protocols and institutions.

The developed method reveals the potential of utilizing DL predictions to estimate confidence scores. This work builds upon existing methodologies while addressing their inherent limitations concerning dataset diversity, inter-institutional variability, and clinical applicability. The developed two-step testing approach underscores the reliability of MC-net’s predictions, bridging the gap between research innovations and clinical applicability. It offers a measure of the network’s reliability, thereby aiding clinicians in making more informed decisions. This approach is particularly advantageous in cases where IDH status determination directly influences decisions in treatment planning, surgical resection, or targeted therapies. The developed framework offers a robust, unbiased tool for noninvasive IDH status determination, enhancing its potential for broader clinical adoption.

## Limitations

Despite the strengths of this study, several limitations warrant consideration. The reliance on multi-contrast MRI may restrict the applicability of the proposed framework in clinical settings where such data are either unavailable or inconsistent in quality. The performance of MC-net has primarily been evaluated on retrospective data, which may not fully capture the diversity and variability encountered in real-world clinical environments. Another limitation is the potential bias introduced by the uneven distribution of IDH status across datasets, which may impact the model’s ability to generalize effectively across different populations.

The use of Bayesian logistic regression (BLR) for confidence estimation involves sampling-based posterior inference, which can be computationally intensive. Optimizing the computational pipeline will be beneficial for facilitating real-time clinical applications. Additionally, the priors employed in the BLR model (eg, informative and non-informative priors) might not fully capture the intricate relationships within the data. Incorporating hierarchical priors could potentially enhance model robustness and flexibility.

The high classification accuracy of MC-net when trained on a large, diverse database highlights the importance of comprehensive training datasets. Expanding the dataset to include a broader range of subjects and acquisition parameters could significantly enhance the model’s performance, generalizability, and robustness, all of which are essential for reliable clinical use. Although the results are promising and demonstrate the potential for clinical implementation, further validation is necessary to establish the framework’s robustness and ensure its successful integration into real-world healthcare workflows.

## Conclusion

We developed a robust DL framework for non-invasive prediction of IDH mutation status in brain gliomas. It includes an MRI-based deep-learning model (MC-net) to predict subject-level IDH status and a Bayesian logistic regression (BLR) model to estimate confidence in the predicted IDH status. The integration of confidence scores enhances the reliability of MC-net’s prediction. The developed framework bridges the gap between DL predictions and their clinical relevance.

Our findings demonstrate that ***MC-net*** achieves high accuracy, precision, and recall across diverse datasets, validating its generalizability and robustness. The comparative evaluation of LR and BLR reveals the superior calibration capabilities of BLR with non-informative priors, as evidenced by the lowest Brier score. This indicates that the inclusion of uncertainty quantification through BLR significantly improves the reliability of confidence scores. The developed framework addresses critical limitations of previous methodologies, including overfitting, limited dataset diversity, and lack of generalizability, making it a strong tool for broader clinical adoption.

By leveraging diverse and externally held-out test data, this approach ensures unbiased evaluation and establishes a reliable method for predicting IDH status and estimating confidence scores. The confidence score represents a tangible measure of the DLN’s reliability in predicting IDH-status. The ability to provide clinicians with IDH status and a confidence score enhances the decision-making process. It allows for a nuanced interpretation of diagnostic results, ensuring that treatment plans are based on confidence in DLN predictions. This framework holds promise for significantly improving patient outcomes by enabling confidence-informed therapeutic strategies in treating gliomas.

## Supplementary Material

vdaf142_suppl_Supplementary_Materials_1

## Data Availability

The study was largely implemented using publicly available databases. Datasets from the collaborator institutions can be made available upon reasonable request.

## Code Availability

The deep learning models were developed using pytorch and the publicly available nnUNet package. Detailed model hyperparameters are described in the supplemental section. The pipeline can be found at https://git.biohpc.swmed.edu/ansir/idh_prediction_pipeline.
