# Supplementary material for: Bridging the clinical gap: Confidence informed IDH prediction in brain gliomas using MRI and deep learning
Source: Neurooncol Adv. 2025 Jul 25;7(1):vdaf142. doi: 10.1093/noajnl/vdaf142 (PMC12365901; doi:10.1093/noajnl/vdaf142)
Supplement: vdaf142_suppl_Supplementary_Materials_1 [file vdaf142_suppl_supplementary_materials_1.docx]

**SUPPLEMENTAL MATERIAL**

**ROC methodology**

The network output maps the tumor voxels as IDH mutated or IDH wild type. For each subject in the test set, the percentage of IDH-mutated voxels was obtained by dividing the predicted number of IDH-mutated voxels by the total number of predicted voxels in each tumor. The percent mutated voxels can be viewed as a network output prediction likelihood of the tumor being IDH-mutated. Our work used majority voting (the 50% threshold) to determine subject-level IDH prediction. For the ROC analysis, the percent of IDH mutated voxels was sorted and used as separate thresholds (cut-points) to determine IDH mutation status for the subjects across the test set for each new cut-point. The resulting predicted IDH class membership was compared to the ground truth values to determine sensitivity (true positive rate) and 1- specificity (false positive rate) at each threshold.  The resulting values were plotted using R Programming to obtain an ROC curve (true positive rate against false positive rate).  Routines in R programming were used to fit the curves and determine the area under the curve (AUC). This procedure was repeated for both different training combinations.


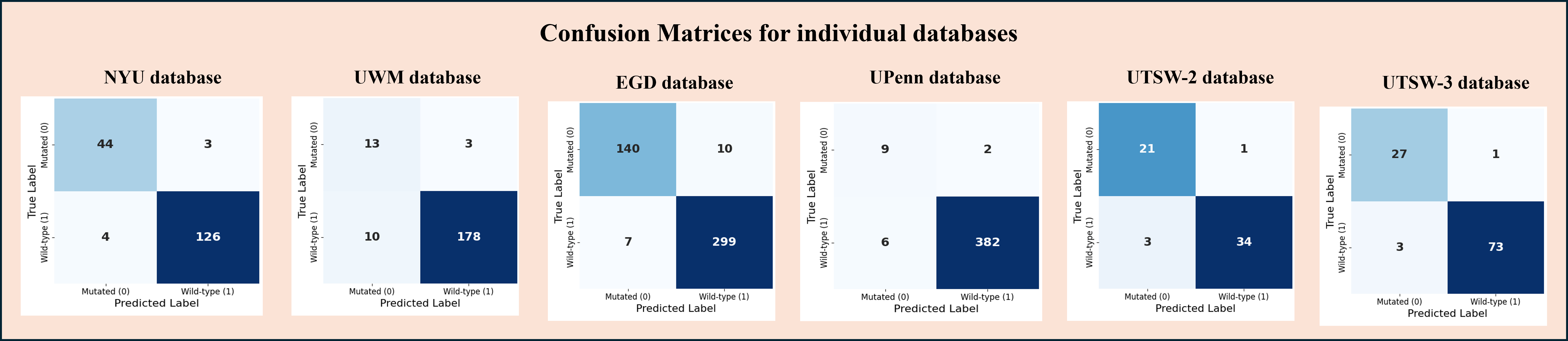


Figure 1: Represents the confusion matrix for each test cohort.

Evaluating the model’s performance on radiologically difficult cases—such as enhancing grade 4 gliomas or non-enhancing lower grade tumors—could offer important insight into real-world applicability. A subgroup analysis on non-enhancing lower grade tumors (Grade 2 & 3) from TG-1 & TG-2 was implemented. We report accuracy, precision, recall, and F1 scores (Table 1).

Table 1: Represents the performance of MC-net on non-enhancing lower grade tumors (Grade 2 & 3) from TG-1 & TG-2.

| **IDH Classification accuracy on** **non-enhancing lower grade tumors (Grade 2 & 3) from TG-1 & TG-2** | | | | | | |
| --- | --- | --- | --- | --- | --- | --- |
| **Training Dataset** | **Metrics** | **NYU (36)** | **EGD (101)** | **UTSW-2 (17)** | **UTSW-3 (17)** | **Overall Accuracy** |
|  |  | (30 / 6) | (88 / 13) | (12 / 5) | (15 / 2) |  |
| **TCIA +**  **UTSW-1 + IvyGAP + UCSF**  1088 Subjects  (301 / 787) | Accuracy | **97.2** | **96.0** | **88.2** | **100.0** | **95.9** |
|  | Accuracy  (IDH-mut class) | 100 | 96.6 | 100 | 100 | 97.9 |
|  | Accuracy  (wildtype class) | 83.3 | 92.3 | 60.0 | 100 | 84.6 |
|  | Precision | 100 | 80 | 100 | 100 | 88 |
|  | Recall | 83.3 | 92.3 | 60 | 100 | 84.6 |
|  | F1-score | 90.9 | 85.7 | 75 | 100 | 86.3 |
|  | AUC | 0.98 | 0.93 | 0.91 | 1.0 | 0.94 |

*Table 2: Hyperparameters used to develop the MC-net.*

| **Training Hyper parameters** |
| --- |
|  |
| "batch_size": "100", |
| "configuration_manager": "{'data_identifier': 'nnUNetPlans_2d', 'preprocessor_name': 'DefaultPreprocessor', 'batch_size': 100, 'patch_size': [192, 160], 'median_image_size_in_voxels': [165.0, 138.0], 'spacing': [1.0, 1.0], 'normalization_schemes': ['ZScoreNormalization', 'ZScoreNormalization', 'ZScoreNormalization', 'ZScoreNormalization'], 'use_mask_for_norm': [True, True, True, True], 'UNet_class_name': 'PlainConvUNet', 'UNet_base_num_features': 32, 'n_conv_per_stage_encoder': [2, 2, 2, 2, 2, 2], 'n_conv_per_stage_decoder': [2, 2, 2, 2, 2], 'num_pool_per_axis': [5, 5], 'pool_op_kernel_sizes': [[1, 1], [2, 2], [2, 2], [2, 2], [2, 2], [2, 2]], 'conv_kernel_sizes': [[3, 3], [3, 3], [3, 3], [3, 3], [3, 3], [3, 3]], 'unet_max_num_features': 512, 'resampling_fn_data': 'resample_data_or_seg_to_shape', 'resampling_fn_seg': 'resample_data_or_seg_to_shape', 'resampling_fn_data_kwargs': {'is_seg': False, 'order': 3, 'order_z': 0, 'force_separate_z': None}, 'resampling_fn_seg_kwargs': {'is_seg': True, 'order': 1, 'order_z': 0, 'force_separate_z': None}, 'resampling_fn_probabilities': 'resample_data_or_seg_to_shape', 'resampling_fn_probabilities_kwargs': {'is_seg': False, 'order': 1, 'order_z': 0, 'force_separate_z': None}, 'batch_dice': True}", |
| "configuration_name": "2d", |
| "cudnn_version": 8700, |
| "current_epoch": "0", |
| "dataloader_train": "<nnunetv2.training.data_augmentation.custom_transforms.limited_length_multithreaded_augmenter.LimitedLenWrapper object at 0x2aac14416c50>", |
| "dataloader_train.generator": "<nnunetv2.training.dataloading.data_loader_2d.nnUNetDataLoader2D object at 0x2aac14417f40>", |
| "dataloader_train.num_processes": "72", |
| "dataloader_train.transform": "Compose ( [SpatialTransform( independent_scale_for_each_axis = True, p_rot_per_sample = 0.4, p_scale_per_sample = 0.2, p_el_per_sample = 0.2, data_key = 'data', label_key = 'seg', patch_size = [192, 160], patch_center_dist_from_border = None, do_elastic_deform = False, alpha = (0.0, 1000.0), sigma = (10.0, 13.0), do_rotation = True, angle_x = (-3.141592653589793, 3.141592653589793), angle_y = (0, 0), angle_z = (0, 0), do_scale = True, scale = (0.7, 1.43), border_mode_data = 'constant', border_cval_data = 0, order_data = 3, border_mode_seg = 'constant', border_cval_seg = -1, order_seg = 1, random_crop = False, p_rot_per_axis = 0.5, p_independent_scale_per_axis = 1 ), OneOfTransform( list_of_transforms = [MedianFilterTransform( p_per_sample = 0.2, p_per_channel = 0.5, data_key = 'data', filter_size = (2, 8), same_for_each_channel = False ), GaussianBlurTransform( p_per_sample = 0.2, different_sigma_per_channel = True, p_per_channel = 0.5, data_key = 'data', blur_sigma = (0.3, 1.5), different_sigma_per_axis = False, p_isotropic = 0 )] ), GaussianNoiseTransform( p_per_sample = 0.1, data_key = 'data', noise_variance = (0, 0.1), p_per_channel = 1, per_channel = False ), BrightnessTransform( p_per_sample = 0.1, data_key = 'data', mu = 0, sigma = 0.5, per_channel = True, p_per_channel = 0.5 ), OneOfTransform( list_of_transforms = [ContrastAugmentationTransform( p_per_sample = 0.2, data_key = 'data', contrast_range = (0.5, 2), preserve_range = True, per_channel = True, p_per_channel = 0.5 ), ContrastAugmentationTransform( p_per_sample = 0.2, data_key = 'data', contrast_range = (0.5, 2), preserve_range = False, per_channel = True, p_per_channel = 0.5 )] ), SimulateLowResolutionTransform( order_upsample = 3, order_downsample = 0, channels = None, per_channel = True, p_per_channel = 0.5, p_per_sample = 0.15, data_key = 'data', zoom_range = (0.25, 1), ignore_axes = None ), GammaTransform( p_per_sample = 0.1, retain_stats = True, per_channel = True, data_key = 'data', gamma_range = (0.7, 1.5), invert_image = True ), GammaTransform( p_per_sample = 0.1, retain_stats = True, per_channel = True, data_key = 'data', gamma_range = (0.7, 1.5), invert_image = True ), MirrorTransform( p_per_sample = 1, data_key = 'data', label_key = 'seg', axes = (0, 1) ), BlankRectangleTransform( rectangle_size = [[19, 64], [16, 53]], num_rectangles = (1, 5), force_square = False, p_per_sample = 0.4, p_per_channel = 0.5, apply_to_keys = ('data',), color_fn = <function BlankRectangleTransform.__init__.<locals>.<lambda> at 0x2aac1869ce50> ), <batchgenerators.transforms.local_transforms.BrightnessGradientAdditiveTransform object at 0x2aac14417c40>, <batchgenerators.transforms.local_transforms.LocalGammaTransform object at 0x2aac144170d0>, SharpeningTransform( p_per_sample = 0.2, p_per_channel = 0.5, data_key = 'data', strength = (0.1, 1), same_for_each_channel = False ), MaskTransform( apply_to_channels = [0, 1, 2, 3], seg_key = 'seg', data_key = 'data', set_outside_to = 0, mask_idx_in_seg = 0 ), RemoveLabelTransform( output_key = 'seg', input_key = 'seg', replace_with = 0, remove_label = -1 ), RenameTransform( delete_old = True, out_key = 'target', in_key = 'seg' ), DownsampleSegForDSTransform2( axes = None, output_key = 'target', input_key = 'target', order = 0, ds_scales = [[1.0, 1.0], [0.5, 0.5], [0.25, 0.25], [0.125, 0.125], [0.0625, 0.0625]] ), NumpyToTensor( keys = ['data', 'target'], cast_to = 'float' )] )", |
| "dataloader_val": "<nnunetv2.training.data_augmentation.custom_transforms.limited_length_multithreaded_augmenter.LimitedLenWrapper object at 0x2aac186aa1d0>", |
| "dataloader_val.generator": "<nnunetv2.training.dataloading.data_loader_2d.nnUNetDataLoader2D object at 0x2aab700d8b20>", |
| "dataloader_val.num_processes": "72", |
| "dataloader_val.transform": "Compose ( [SpatialTransform( independent_scale_for_each_axis = True, p_rot_per_sample = 0.4, p_scale_per_sample = 0.2, p_el_per_sample = 0.2, data_key = 'data', label_key = 'seg', patch_size = [192, 160], patch_center_dist_from_border = None, do_elastic_deform = False, alpha = (0.0, 1000.0), sigma = (10.0, 13.0), do_rotation = True, angle_x = (-3.141592653589793, 3.141592653589793), angle_y = (0, 0), angle_z = (0, 0), do_scale = True, scale = (0.7, 1.43), border_mode_data = 'constant', border_cval_data = 0, order_data = 3, border_mode_seg = 'constant', border_cval_seg = -1, order_seg = 1, random_crop = False, p_rot_per_axis = 0.5, p_independent_scale_per_axis = 1 ), OneOfTransform( list_of_transforms = [MedianFilterTransform( p_per_sample = 0.2, p_per_channel = 0.5, data_key = 'data', filter_size = (2, 8), same_for_each_channel = False ), GaussianBlurTransform( p_per_sample = 0.2, different_sigma_per_channel = True, p_per_channel = 0.5, data_key = 'data', blur_sigma = (0.3, 1.5), different_sigma_per_axis = False, p_isotropic = 0 )] ), GaussianNoiseTransform( p_per_sample = 0.1, data_key = 'data', noise_variance = (0, 0.1), p_per_channel = 1, per_channel = False ), BrightnessTransform( p_per_sample = 0.1, data_key = 'data', mu = 0, sigma = 0.5, per_channel = True, p_per_channel = 0.5 ), OneOfTransform( list_of_transforms = [ContrastAugmentationTransform( p_per_sample = 0.2, data_key = 'data', contrast_range = (0.5, 2), preserve_range = True, per_channel = True, p_per_channel = 0.5 ), ContrastAugmentationTransform( p_per_sample = 0.2, data_key = 'data', contrast_range = (0.5, 2), preserve_range = False, per_channel = True, p_per_channel = 0.5 )] ), SimulateLowResolutionTransform( order_upsample = 3, order_downsample = 0, channels = None, per_channel = True, p_per_channel = 0.5, p_per_sample = 0.15, data_key = 'data', zoom_range = (0.25, 1), ignore_axes = None ), GammaTransform( p_per_sample = 0.1, retain_stats = True, per_channel = True, data_key = 'data', gamma_range = (0.7, 1.5), invert_image = True ), GammaTransform( p_per_sample = 0.1, retain_stats = True, per_channel = True, data_key = 'data', gamma_range = (0.7, 1.5), invert_image = True ), MirrorTransform( p_per_sample = 1, data_key = 'data', label_key = 'seg', axes = (0, 1) ), BlankRectangleTransform( rectangle_size = [[19, 64], [16, 53]], num_rectangles = (1, 5), force_square = False, p_per_sample = 0.4, p_per_channel = 0.5, apply_to_keys = ('data',), color_fn = <function BlankRectangleTransform.__init__.<locals>.<lambda> at 0x2aac1869ce50> ), <batchgenerators.transforms.local_transforms.BrightnessGradientAdditiveTransform object at 0x2aac14417c40>, <batchgenerators.transforms.local_transforms.LocalGammaTransform object at 0x2aac144170d0>, SharpeningTransform( p_per_sample = 0.2, p_per_channel = 0.5, data_key = 'data', strength = (0.1, 1), same_for_each_channel = False ), MaskTransform( apply_to_channels = [0, 1, 2, 3], seg_key = 'seg', data_key = 'data', set_outside_to = 0, mask_idx_in_seg = 0 ), RemoveLabelTransform( output_key = 'seg', input_key = 'seg', replace_with = 0, remove_label = -1 ), RenameTransform( delete_old = True, out_key = 'target', in_key = 'seg' ), DownsampleSegForDSTransform2( axes = None, output_key = 'target', input_key = 'target', order = 0, ds_scales = [[1.0, 1.0], [0.5, 0.5], [0.25, 0.25], [0.125, 0.125], [0.0625, 0.0625]] ), NumpyToTensor( keys = ['data', 'target'], cast_to = 'float' )] )", |
| "dataset_json": "{'channel_names': {'T1': 0, 'T1C': 1, 'T2': 2, 'Flair': 3}, 'labels': {'background': 0, 'mutated': 1, 'wild-type': 2}, 'numTraining': 1688, 'file_ending': '.nii.gz', 'regions_class_order': 'TCIA, UTSW and UCSF MCon data --> FeTS002 SS and with N4 and ImagePermutation'}", |
| "device": "cuda:0", |
| "disable_checkpointing": "False", |
| "gpu_name": "NVIDIA A100-PCIE-40GB", |
| "grad_scaler": "<torch.cuda.amp.grad_scaler.GradScaler object at 0x2aac143d7e80>", |
| "inference_allowed_mirroring_axes": "(0, 1)", |
| "initial_lr": "0.01", |
| "is_cascaded": "False", |
| "is_ddp": "False", |
| "label_manager": "<nnunetv2.utilities.label_handling.label_handling.LabelManager object at 0x2aaab2326d70>", |
| "logger": "<nnunetv2.training.logging.nnunet_logger.nnUNetLogger object at 0x2aac143d7a90>", |
| "loss": "DeepSupervisionWrapper(\n  (loss): DC_and_CE_loss(\n    (ce): RobustCrossEntropyLoss()\n    (dc): MemoryEfficientSoftDiceLoss()\n  )\n)", |
| "lr_scheduler": "<nnunetv2.training.lr_scheduler.polylr.PolyLRScheduler object at 0x2aac143d7a00>", |
| "my_init_kwargs": "{'plans': {'plans_name': 'nnUNetPlans', 'original_median_spacing_after_transp': [1.0, 1.0, 1.0], 'original_median_shape_after_transp': [142, 165, 138], 'image_reader_writer': 'SimpleITKIO', 'transpose_forward': [0, 1, 2], 'transpose_backward': [0, 1, 2], 'configurations': {'2d': {'data_identifier': 'nnUNetPlans_2d', 'preprocessor_name': 'DefaultPreprocessor', 'batch_size': 100, 'patch_size': [192, 160], 'median_image_size_in_voxels': [165.0, 138.0], 'spacing': [1.0, 1.0], 'normalization_schemes': ['ZScoreNormalization', 'ZScoreNormalization', 'ZScoreNormalization', 'ZScoreNormalization'], 'use_mask_for_norm': [True, True, True, True], 'UNet_class_name': 'PlainConvUNet', 'UNet_base_num_features': 32, 'n_conv_per_stage_encoder': [2, 2, 2, 2, 2, 2], 'n_conv_per_stage_decoder': [2, 2, 2, 2, 2], 'num_pool_per_axis': [5, 5], 'pool_op_kernel_sizes': [[1, 1], [2, 2], [2, 2], [2, 2], [2, 2], [2, 2]], 'conv_kernel_sizes': [[3, 3], [3, 3], [3, 3], [3, 3], [3, 3], [3, 3]], 'unet_max_num_features': 512, 'resampling_fn_data': 'resample_data_or_seg_to_shape', 'resampling_fn_seg': 'resample_data_or_seg_to_shape', 'resampling_fn_data_kwargs': {'is_seg': False, 'order': 3, 'order_z': 0, 'force_separate_z': None}, 'resampling_fn_seg_kwargs': {'is_seg': True, 'order': 1, 'order_z': 0, 'force_separate_z': None}, 'resampling_fn_probabilities': 'resample_data_or_seg_to_shape', 'resampling_fn_probabilities_kwargs': {'is_seg': False, 'order': 1, 'order_z': 0, 'force_separate_z': None}, 'batch_dice': True}, '3d_fullres': {'data_identifier': 'nnUNetPlans_3d_fullres', 'preprocessor_name': 'DefaultPreprocessor', 'batch_size': 4, 'patch_size': [160, 192, 160], 'median_image_size_in_voxels': [142.0, 165.0, 138.0], 'spacing': [1.0, 1.0, 1.0], 'normalization_schemes': ['ZScoreNormalization', 'ZScoreNormalization', 'ZScoreNormalization', 'ZScoreNormalization'], 'use_mask_for_norm': [True, True, True, True], 'UNet_class_name': 'PlainConvUNet', 'UNet_base_num_features': 32, 'n_conv_per_stage_encoder': [2, 2, 2, 2, 2, 2], 'n_conv_per_stage_decoder': [2, 2, 2, 2, 2], 'num_pool_per_axis': [5, 5, 5], 'pool_op_kernel_sizes': [[1, 1, 1], [2, 2, 2], [2, 2, 2], [2, 2, 2], [2, 2, 2], [2, 2, 2]], 'conv_kernel_sizes': [[3, 3, 3], [3, 3, 3], [3, 3, 3], [3, 3, 3], [3, 3, 3], [3, 3, 3]], 'unet_max_num_features': 320, 'resampling_fn_data': 'resample_data_or_seg_to_shape', 'resampling_fn_seg': 'resample_data_or_seg_to_shape', 'resampling_fn_data_kwargs': {'is_seg': False, 'order': 3, 'order_z': 0, 'force_separate_z': None}, 'resampling_fn_seg_kwargs': {'is_seg': True, 'order': 1, 'order_z': 0, 'force_separate_z': None}, 'resampling_fn_probabilities': 'resample_data_or_seg_to_shape', 'resampling_fn_probabilities_kwargs': {'is_seg': False, 'order': 1, 'order_z': 0, 'force_separate_z': None}, 'batch_dice': False}}, 'experiment_planner_used': 'ExperimentPlanner', 'label_manager': 'LabelManager'}", |
| "network": "PlainConvUNet", |
| "num_epochs": "1000", |
| "num_input_channels": "4", |
| "num_iterations_per_epoch": "250", |
| "num_val_iterations_per_epoch": "50", |
| "optimizer": "SGD (\nParameter Group 0\n    dampening: 0\n    differentiable: False\n    foreach: None\n    initial_lr: 0.01\n    lr: 0.01\n    maximize: False\n    momentum: 0.99\n    nesterov: True\n    weight_decay: 3e-05\n)", |
| "oversample_foreground_percent": "0.33", |
